# Autism-Associated Gut Microbiota–Derived *Enterococcus facium* Modulates Gut–Brain Axis Function and Behavior in Mice

**DOI:** 10.3390/pathogens14121191

**Published:** 2025-11-21

**Authors:** Renzhen Ma, Hidayat Ullah, Fatemeh Shahbazi Bohlooli, Yuqi Wen, Yi Xin, Jiayi Wang, Shuming Lu, Liang Wang

**Affiliations:** 1 Department of Biotechnology, College of Basic Medical Science, Dalian Medical University, Dalian 116011, Chinahidayat.khan89@yahoo.com (H.U.);; 2 Guangdong Provincial Key Laboratory of Natural Drugs Research and Development, Guangdong Medical University, Dongguan 523808, China; 3 International Education College, Dalian Medical University, Dalian 116011, China; 4 National Joint Engineering Laboratory, The First Affiliated Hospital of Dalian Medical University, Dalian 116011, China; 5 Department of Gastroenterology, First Affiliated Hospital of Dalian Medical University, Dalian 116011, China

**Keywords:** behavioral changes, *Enterococcus faecium*, gut barrier function, gut microbiota, gut–brain communication

## Abstract

Autism spectrum disorder (ASD) is a complex neurodevelopmental condition characterized by social communication deficits, repetitive behaviors, and restricted interests. Although its pathogenesis is not fully understood, emerging evidence suggests a connection between gut microbiota alterations and ASD. The role of specific bacterial species, particularly *Enterococcus faecium*, in the development of ASD remains unclear. This study aimed to investigate the impact of *E. faecium* derived from the feces of autistic children on mice. Thirty male BALB/c mice were divided into three groups: control, *E. coli*, and *E. faecium* treatment groups. *E. faecium* was administered orally for 30 days. Behavioral assessments, including open field tests, sucrose preference, Y-maze, and social interaction tests, were performed to evaluate anxiety, depression, memory, and social behavior. Additionally, serum 5-HT levels were measured, and colon and brain tissues were analyzed for inflammation, blood–brain barrier (BBB) integrity, and histological changes. Stool DNA sequencing was used to assess microbiota diversity and composition. Treatment with *E. faecium* significantly altered behavior in mice, including increased anxiety, depression, impaired memory, and social dysfunction. Colon histology revealed severe damage, including increased inflammation, reduced tight junction protein expression, and decreased mucin-2 levels. Elevated serum lipopolysaccharide (LPS) levels indicated systemic inflammation, and gut microbiota analysis showed significant dysbiosis. In the brain, particularly within the hippocampus and cortical regions, *E. faecium* induced neural damage, heightened inflammation, and compromised blood–brain barrier integrity. *Enterococcus faecium* from autistic patients can induce significant behavioral changes in mice, potentially via gut microbiota dysbiosis, intestinal barrier disruption, and brain inflammation. These findings suggest that *E. faecium* may contribute to gut–brain axis dysregulation in ASD, although further mechanistic studies are warranted.

## 1. Introduction

Autism spectrum disorder (ASD) is usually diagnosed in early childhood, typically during the preschool years, with an estimated global prevalence of around 1% [1]. ASD is characterized by social communication impairments, restricted interests, and repetitive behaviors, with comorbidities observed in about 70% of individuals [2]. Gastrointestinal (GI) complications, such as constipation, diarrhea, and abdominal pain, are prevalent in ASD, and these symptoms are closely linked to gut microbiota imbalance [3]. An imbalance in gut microbiota can impair normal gastrointestinal function, leading to digestive system problems that worsen ASD-related gastrointestinal discomfort and, in turn, may exacerbate behavioral symptoms. Additionally, gut dysbiosis may influence the gut–brain axis (GBA), affecting the gut nervous system, neuroendocrine system, and immune system, which can directly modulate brain function and behavior [4,5]. Studies found that gut microbiota from ASD individuals can induce behavioral alterations and symptoms in animal models, suggesting a strong connection between microbiota composition and ASD manifestation [6]. The microbiota composition in ASD patients differs from that in neurotypical individuals, with an increased abundance of *Proteobacteria*, *Firmicutes*, *Clostridia*, and *Lactobacillus* species, while *Bacteroidetes*, *Actinobacteria*, and *Akkermansia* species are underrepresented [7,8]. Despite these findings, no single highly specific and sensitive biomarker has yet been identified for the diagnosis of ASD, and further research is needed to verify the reliability and clinical application of ASD-related biomarkers [9,10,11].

The blood–brain barrier (BBB) is composed of tight junctions between endothelial cells and serves as a selective semipermeable membrane crucial for maintaining brain homeostasis. Neuroinflammation frequently disrupts the integrity of the blood–brain barrier, playing a key role in the development and progression of neurodevelopmental disorders [12,13]. Studies suggest that low-dose penicillin treatments during late pregnancy or early postnatal periods can affect BBB integrity by increasing the expression of pro-inflammatory cytokines in the brain [14,15]. *Enterococcus faecium*, a Gram-positive bacterium in the gut microbiota, is an opportunistic pathogen that can cause infections in immunocompromised individuals. It is known for its natural resistance to different antibiotics, including vancomycin, complicating treatment [16,17]. Furthermore, *E. faecium* isolated from individuals with inflammatory conditions has been shown to promote the onset of colitis, highlighting its potential role in gut-related disorders [18].

Based on previous findings, we isolated the *E. faecium* from the feces of children with ASD and utilized a mouse model to investigate its effects on behavioral changes, brain tissue, gut microbiota, and the intestinal barrier. This study aimed to explore the role of *E. faecium* in ASD pathophysiology and to assess whether its abundance could serve as a potential biomarker, offering new opportunities for the early diagnosis and treatment of ASD.

## 2. Materials and Methods

### 2.1. Chemicals and Reagents

The main experimental reagents used in this study include brain-heart infusion broth medium from Qingdao Yuanhai Biological Technology Co., Ltd., Qingdao, China; xylene from Tianjin Damao Chemical Reagent Factory, Tianjin, China, PBS powder; citric acid antigen buffer repair powder from Beijing Solarbio Technology Co., Ltd., Tianjin, China, and 4% paraformaldehyde from Seven Innovation (Beijing) Biotechnology Co., Ltd., Beijing, China. The HiScriptIII All-in-one RT Super Mix for qPCR and Cham Q Universal SYBR qPCR Master Mix were purchased from Nanjing Novozan Biotechnology Co., Ltd., Nanjing, China. Key antibodies, including MUC2, Occludin, Claudin 5, β-Actin, ZO-1, and Claudin 1 rabbit polyclonal antibodies, were sourced from Proteintech. ELISA kits for mouse IL-6, IL-10, 5-HT, and LPS were obtained from Jianglai Biological. The DAB color development kit (20×) was from Beijing ZhongShan Golden Bridge Biotechnology Co., Ltd., Beijing, China, and the fecal genomic DNA extraction kits were from Fuji Biological Co., Ltd., Tokyo, Japan. 

### 2.2. Animal Housing

A total of 30 male BALB/c mice, weighing approximately 13–14 g and aged 3 weeks, were purchased from the SPF facility of Dalian Medical University and used in the experiment with proper ethical approval from the ethical committee under approval number 202310247. The mice were housed in standard laboratory conditions, adhering to a 12 h light/dark cycle. The animals had free access to food and water throughout the acclimation period. After acclimatization, mice were randomly assigned to each group using a random number generator to minimize selection bias. Three groups, each containing 10 mice. The groups include the normal control group, the *Escherichia coli* group, and the *Enterococcus faecium* group. The mice were labeled, weighed, and recorded accordingly.

### 2.3. Isolation and Identification of Enterococcus faecium

*Enterococcus faecium* was isolated from fecal samples collected from six children (aged 4–9 years) clinically diagnosed with autism spectrum disorder (ASD) in the Dalian region, with written informed consent obtained from their parents or legal guardians. The *Escherichia coli* strain used as a control was a standard non-pathogenic laboratory strain obtained from the American Type Culture Collection (ATCC 25922). The strain was cultured in Luria–Bertani (LB) medium at 37 °C and used as a bacterial control for comparison with *Enterococcus faecium*. All fecal samples were processed individually under sterile conditions, and bacterial isolates were designated as Bac strains. Raw sequencing data obtained from next-generation sequencing were base-called, quality-filtered, and assembled into high-confidence bacterial sequences for downstream taxonomic and genomic analyses.

### 2.4. Establishment and Application of the Standard Curve

Bacterial cultures were prepared by isolating a single colony of the bacterial strain and inoculating it into a brain heart infusion broth, followed by overnight incubation. The culture was then transferred to a shaking incubator for the next 6 h to prepare the seed cultures. Different dilutions of the culture were made and prepared, and absorbance at 589 nm was measured, ensuring that the values remained below 1.0. The colony count was determined by plating the dilution onto nutrient agar plates and incubating them overnight. Each dilution was plated in triplicate, and colony numbers were recorded. A standard curve was then constructed by plotting absorbance value against the corresponding colony to establish a linear relationship. This standard curve was subsequently used to calculate the corresponding absorbance values for the desired bacterial count.

### 2.5. Administration of Enterococcus faecium and Study Protocol

To explore the pathogenesis of *Enterococcus faecium*, the animal model was established by administering the bacterial suspension containing 1 × 10^9^ CFU. Briefly, the suspension was centrifuged at 5000 rpm for 10 min, and the bacteria were collected, followed by washing with normal saline and resuspended in normal saline to ensure a concentration of 1.0 × 10^9^ CFU per 0.2 mL of PBS solution for use. Both groups, *Escherichia coli* group and *Enterococcus faecium*, received and gavaged with 0.2 mL of their respective bacterial dilution daily at the same time; meanwhile, the normal control group received the same volume of saline. Body weight, behavioral tests, and other parameters were recorded accordingly throughout experimental periods. The administration was continued for 30 days. Before the scarification of the mice, stool samples were collected and stored at −80 °C for future analysis. The mice were euthanized by cervical dislocation. Peripheral blood was collected via eyeball puncture, left at room temperature for 1 h, and then centrifuged at 3000 rpm for 10 min to collect the serum. A portion of colon tissue was fixed in 4% paraformaldehyde, and the remainder was stored at −80 °C. Half of the brain was fixed in 4% paraformaldehyde, while the hippocampus and cortex were isolated and stored at −80 °C for further analysis.

### 2.6. Open Field Test

All behavioral assessments were conducted sequentially over consecutive days to minimize stress-induced interference, following the order: open field, sucrose preference, Y-maze, and three-chamber social interaction tests. Each behavioral test was performed at the same time of day with a 24 h interval between sessions to allow adequate recovery and to avoid carryover effects. Throughout all behavioral experiments, mice were identified only by coded labels, and data collection and video scoring were performed by independent investigators blinded to the treatment groups. The open field test was used to assess the locomotor activity and anxiety-like behavior of the mice. Mice were placed in a novel open-field arena, and their exploratory behavior was recorded by measuring the total distance traveled and the time spent in the central area over 5 min. After each trial, mice were transferred to a clean cage to prevent interaction with other animals and avoid influencing subsequent tests. The open field chamber was wiped with 75% alcohol to remove any residual odors before testing the next mouse, allowing the alcohol to evaporate before starting the next trial.

### 2.7. Sucrose Preference Experiment

The mice were individually housed in a single cage for 48 h, with access to both 1% sucrose water and regular drinking water. This test was performed 24 h after the open field test to allow recovery and prevent behavioral carryover effects. The positions of the two bottles were switched after 24 h. At the end of the adaptation period, the mice were water-deprived for 24 h. Following this, the volume of water consumed from both bottles was measured within hours, with the bottles exchanged after 12 h. was measured. Sucrose preferences were calculated using the following formula: Sucrose preference (%) = (sucrose water intake/(sucrose water intake + drinking water intake)) × 100%

### 2.8. Y Maze Test

The Y maze consists of three opaque arms (Labeled A, B, and C) arranged at 120° angles. The Y-maze test was conducted one day after the sucrose preference test, and all scoring was performed under blinded conditions. Mice are gently placed at the end of arm A and allowed to explore freely for 5 min. the sequence in which the mice enter the area is recorded, with full entry of limbs into an arm considered a valid entry. After each trial, the maze was cleaned with 75% alcohol, and the alcohol was allowed to evaporate before testing the next mouse. The spontaneous alternation rate is calculated as follows:

spontaneous alternating rate (%) = [(spontaneous alternation)/(total number of arm advances − 2)] ×100.

### 2.9. Three-Box Social

To further explore the social behavior of the mice, the social preference test was conducted. The three-chamber social test was performed after completion of the Y-maze, following a 24 h recovery interval, and behavioral analysis was conducted by blinded investigators using coded identifiers. The experimental setup consists of three-chambered boxes (62 cm × 42 cm × 24 cm) and two cylindrical cages with diameters of 8 cm. During the adaptation period, experimental mice were placed in the boxes for free exploration for 5 min. in the testing phase, an unfamiliar “tool” mouse (matched by sex and strain to the experimental mouse) was placed in one of the cylindrical cages, while an empty cylindrical cage was placed in the other. The mouse was then allowed to move freely within the three chambers for 10 min. the duration of direct contact between the test mouse and either the tool mouse or the empty cage was recorded. After each trial, the apparatus was cleaned with 75% alcohol. The social preference of the mice was evaluated using the social index, calculated as:

Social Index = Social Time/(Social Time + Exploration Time).

### 2.10. Histopathological Examination

Colon and brain tissues were fixed in 4% paraformaldehyde for 48 h, dehydrated through graded ethanol solutions, and cleared in xylene. After embedding in paraffin wax at 62 °C for 2 h, the tissues were sectioned into 3 μm slices using a microtome. The sections were then placed in warm water, transferred to glass slides, and dried in a 100 °C oven. For hematoxylin staining, the sections were placed in hematoxylin solution for 3 min, rinsed with tap water, and differentiated with 1% acid ethanol for 1 s, followed by a 10 min rinse in tap water. Eosin counterstaining was performed for 30 s. The sections were dehydrated through a series of ethanol and xylene solutions and then mounted with neutral gum after air-drying. Finally, the sections were observed under a microscope. Furthermore, Alcian Blue-Periodic Acid-Schiff (AB-PAS) staining was employed to detect goblet cells. Then, they were stained with Alcian Blue for 8 min, washed with water for 10 min, and treated with periodic acid for 5 min, followed by a 10 min rinse. Schiff’s reagent was applied for 5 min in the dark and then rinsed again. All tissue sampling, histological preparation, and quantitative scoring were performed under blinded conditions by two independent observers using coded slides. The staining procedures and quantification methods followed previously established protocols for intestinal and brain histopathology [19,20], ensuring methodological reliability and reproducibility.

### 2.11. Immunohistochemistry

For immunohistochemical staining, tissue sections were dewaxed, antigen retrieval was performed using citrate buffer, and endogenous peroxidase was blocked. After blocking non-specific binding, sections were incubated overnight at 4 °C with a primary antibody (Iba-1, 1:200), followed by incubation with a biotinylated secondary antibody. HRP-conjugated streptavidin and DAB were used for visualization, with counterstaining in hematoxylin. For immunofluorescence, after antigen retrieval and blocking with 5% BSA, primary and fluorescent secondary antibodies were applied, followed by DAPI staining and mounting with an anti-fade medium for fluorescence microscopy. All immunohistochemical analyses were conducted under blinded conditions, and coded slides were evaluated independently by two observers. Quantitative assessment of positive staining intensity and area was performed using ImageJ software (ImageJ2) version 2.9.0. to minimize subjective bias.

### 2.12. Fecal DNA Extraction and 16S rRNA Sequencing

DNA from fecal samples was extracted using the FOREGENE stool DNA extraction kit following the manufacturer’s instructions. The 16S rDNA region was then amplified using the primers GC-341F (5′-CGC CCG GGG CGC GCC CCG GGC GGG GCG GGG GCA CGG GGG G-CCT ACG GGA GGC AGC AG-3′) and 805R (5′-GAC TAC HVG GGT ATC TAA TCC-3′) for bacterial analysis. For 16S rDNA high-throughput sequencing, the Illumina Miseq™/Hiseq™ platform was utilized. Raw sequence data were processed to remove barcode, primer, and adapter sequences, with paired-end reads assembled based on overlap. Barcode tags were used for sample identification, and the resulting data underwent quality control to ensure valid and accurate sequencing results.

### 2.13. Western Blotting

The total proteins from colon and cortical tissues were extracted using RIPA lysis buffer supplemented with PMSF, followed by homogenization and centrifugation. The protein concentration was measured using a Nanodrop. The proteins were then separated using SDS-PAGE, transferred to PVDF membranes, and blocked with skimmed milk. After incubation with primary and secondary antibodies, protein expression was detected using an enhanced chemiluminescence (ECL) kit, and the images were captured for analysis.

### 2.14. mRNA Expression Level Measurement by RT-qPCR

RNA was extracted from colon tissues using the Trizol method. The tissues were homogenized in cold Trizol, followed by chloroform and isopropanol extraction to precipitate the RNA. The RNA was then washed with 75% ethanol and resuspended in DEPC-treated water. RNA concentration was measured using Nanodrop and adjusted to approximately 1000 μg/μL for storage. For RT-PCR, cDNA was synthesized from RNA using a reverse transcription reaction, and the expression of TNF-α and IL-6 in the colon tissue was analyzed by PCR.

### 2.15. Measurement of Cytokine Level in the Colon by ELISA

ELISA was used to detect LPS and 5-HT levels in serum, and TNF-α and IL-6 expression in hippocampal tissue. Samples were processed and incubated with specific antibodies and substrate solutions according to the kit’s instructions, followed by measurement of optical density (OD) at 450 nm.

### 2.16. Statistical Analysis

Statistical analyses were performed using GraphPad Prism 9.0 software. Data are presented as mean ± standard deviation (SD). Normality of data distribution was verified using the Shapiro–Wilk test, and all datasets met the assumptions required for parametric analysis. Group comparisons were analyzed using one-way analysis of variance (ANOVA) followed by Tukey’s post hoc test for multiple-comparison correction.

## 3. Results

### 3.1. Bacterial Strain Sequencing

**Figure 1:**
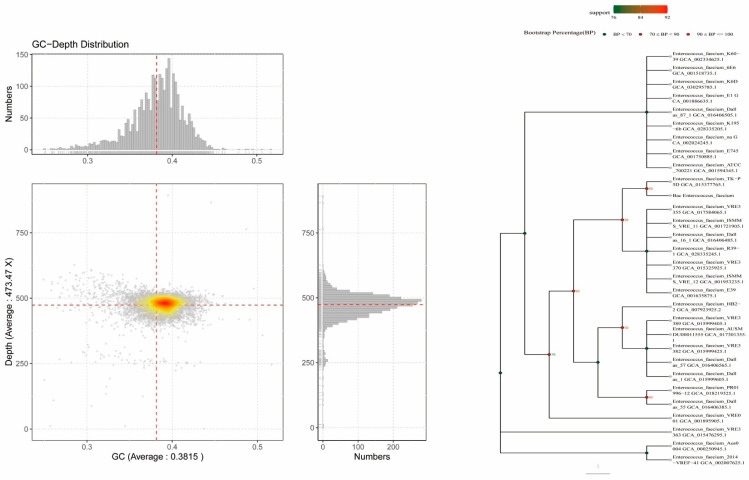
The genomic characteristics and phylogenetic analysis of the bacterial strain. Genomic GC-depth distribution map and 16S rRNA-based evolutionary tree.

The sequencing results of the bacterial genome map revealed that the scatter points were predominantly distributed within the GC content range of approximately 38% with no significant culturing of foreign sequences. These results indicate good genomic integrity and the absence of contamination from exogenous species. A BLAST search was employed to compare the predicted 16S rRNA sequences with the NCBI 16S database. Phylogenetic analysis based on this comparison identified the bacterium as *Enterococcus faecium*, as shown in Figure 1.

### 3.2. Standard Curve

**Figure 2:**
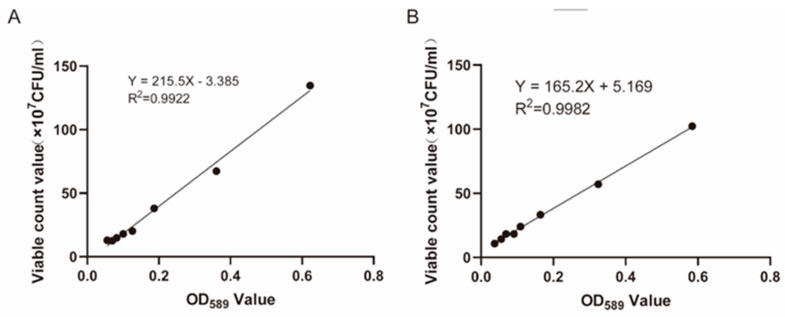
The standard curve between the viable count value and the absorbance value. (**A**) *Enterococcus faecium* (**B**) *Escherichia coli*.

The absorbance values of *Escherichia coli* and *Enterococcus faecium* were correlated with bacterial concentrations in the suspension within a specific concentration range, exhibiting a strong linear relationship. At an absorbance of 589 nm, the absorbance values of the bacterial suspensions of *E. coli* and *E. faecium* were plotted on the x-axis, while the bacterial plate count results (×10^7^ CFU/mL) were plotted on the y-axis. Standard curves for both bacteria were established to determine the relationship between bacterial counts and absorbance values. The regression equation for *E. faecium* was Y = 215.5X − 3.385 (R^2^ = 0.9922) (Figure 2A), and for *E. coli*, the regression equation was Y = 165.2X + 5.169 (R^2^ = 0.9982).

### 3.3. Behavioral Alterations Induced by E. faecium Administration

**Figure 3:**
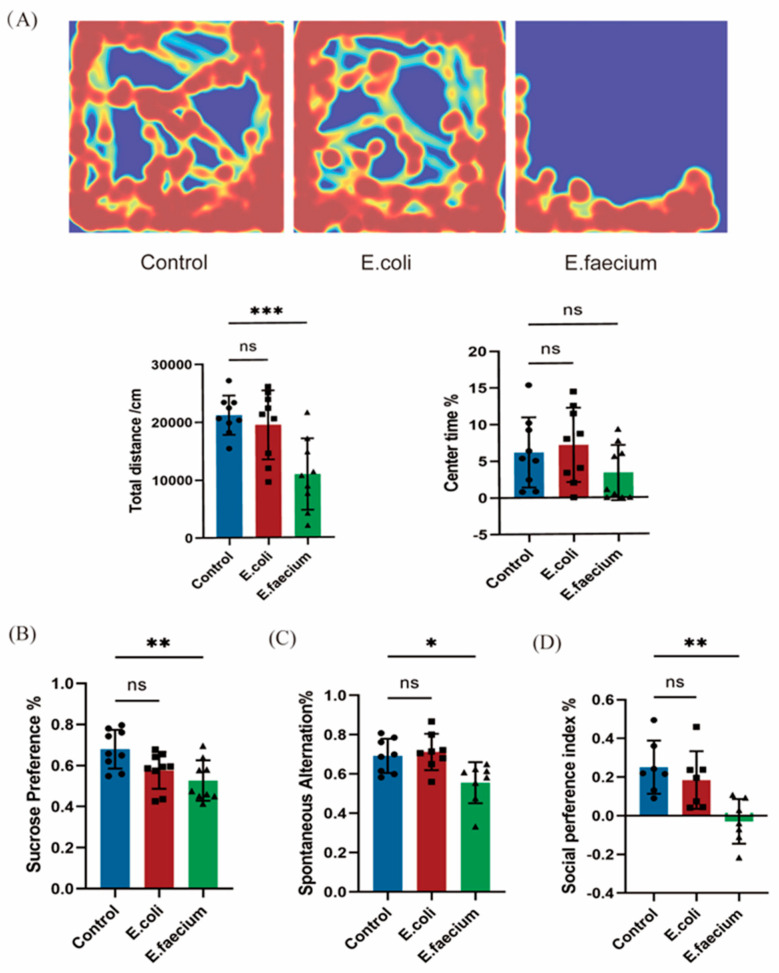
Behavioral alteration following *E. faecium* and *E. coli* administration (**A**) In the open-field test, the *E. faecium* group traveled a shorter distance and spent less time in the center compared to the Control group. (**B**) The *E. faecium* group showed a significantly reduced sugar water preference. (**C**) The *E. faecium* group exhibited impaired memory with decreased spontaneous alternations in the Y-maze test. (**D**) The *E. faecium* group had a significant reduction in the social preference index, indicating decreased sociability. * *p* < 0.05, ** *p* < 0.01, *** *p* < 0.001; ns, not significant.

The behavioral test results showed distinct patterns across the groups. In the open-field test (Figure 3A), mice in the Control and *E. coli* groups were more active and moved around the arena, while the *E. faecium* group tended to cluster in one corner. The total distance traveled by the *E. faecium* group was significantly lower compared to the Control group, although no significant difference was found between the *E. coli* and Control groups. There was also a trend towards less time spent in the central area for the *E. faecium* group, but this difference was not statistically significant, and the time spent in the central area by the *E. coli* group mice remained unchanged. In the sugar water preference test (Figure 3B), the *E. faecium* group showed a significantly reduced preference for sugar water compared to the Control group, while the *E. coli* group exhibited a slight, non-significant decrease. The Y-maze test, which measures short-term memory, revealed a significant decrease in spontaneous alternations in the *E. faecium* group, suggesting impaired memory function compared to the Control group. In contrast, the *E. coli* group showed no significant change in spontaneous alteration behavior (Figure 3C). Finally, in the three-chamber social test (Figure 3D), the *E. faecium* group exhibited a significant reduction in the social preference index, indicating a decrease in sociability, while no changes were observed in the *E. coli* group.

### 3.4. Gut Microbiota Alterations by E. faecium Administration

**Figure 4:**
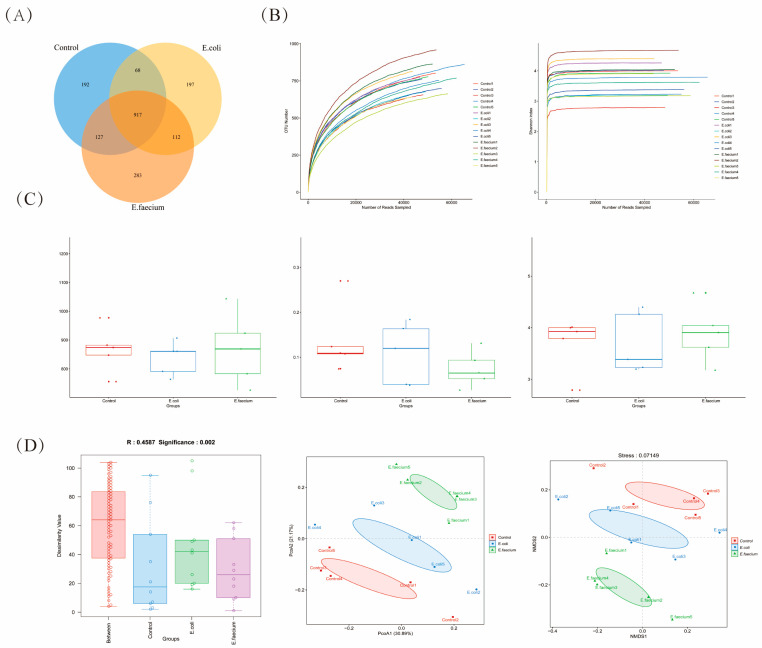
Effects of *E. faecium* administration on gut microbiota composition and diversity. (**A**) Venn diagram showing shared and unique OTUs among Control, *E. coli*, and *E. faecium* groups. (**B**) Rarefaction curves assessing sequencing depth based on observed species and Shannon diversity. (**C**) Alpha diversity indices (Chao1, Shannon, Simpson) comparing richness and diversity across groups. (**D**) β-diversity analysis using ANOSIM (R = 0.4587, *p* = 0.002), PCoA, and NMDS plots indicating significant microbiota structure differences among groups.

To further assess the effect of *E. faecium* administration on the gut microbiota in the mouse model, 16S rRNA sequencing was prominently utilized. The OUT-clustering analysis was performed by grouping effective sequences with >97% similarity into an OUT. A Venn diagram was used to visualize the OUT-distribution differences between the three experimental groups. The results showed that the control group had 1292 OTUs, and the E. faecium group had 1439 OTUs. A total of 917 OTUs were shared by all three groups. The control group had 192 unique OTUs, the E coli. The group showed 197, and the *E. faecium* group had 283 unique OTUs. Notably, the *E. faecium* group exhibited a significantly higher number of unique OTUs compared to both the Control and *E. coli* groups, as depicted in Figure 4A.

Alpha diversity parameters were employed to assess the microbial diversity within each sample. Specifically, the Chao. Shannon and Simpson indices were applied to evaluate different aspects of the microbiome. The Chao index measures microbial abundances, with a higher value indicating greater bacterial richness. the Shannon index reflects microbial diversity, with a higher score indicating a more diverse species composition. While the Simpson index assesses the concentration of dominant species, where lower values suggest a more even distribution of species. The sequencing results revealed in Figure 4B that *E. faecium* showed a similar Chao1 index as that of the control group, while the E. coli group exhibited a decreased Chao1 index. Additionally, the Simpson index in the *E. faecium* group showed a slight decrease, and the Shannon index in the *E. coli* group showed a small decrease, although neither of these differences was statistically significant. Furthermore, β-diversity analysis was conducted, and ANOSIM was performed, with results indicating that R = 0.4587 and *p* = 0.002, which suggests significant between-group differences. PCoA analysis, based on Bray–Curtis distances, demonstrated that PC1 explained 30.89% of the variability in microbiota composition, while PC2 explained 21.17%. The PCoA2 axis effectively differentiated the microbiota structures of the Control, *E. coli*, and *E. faecium* groups. The NMDS analysis further confirmed the distinct differences in microbiota composition among the three groups (Figure 4C,D).

### 3.5. E. faecium Alters Gut Microbiota at Different Taxonomic Levels

**Figure 5:**
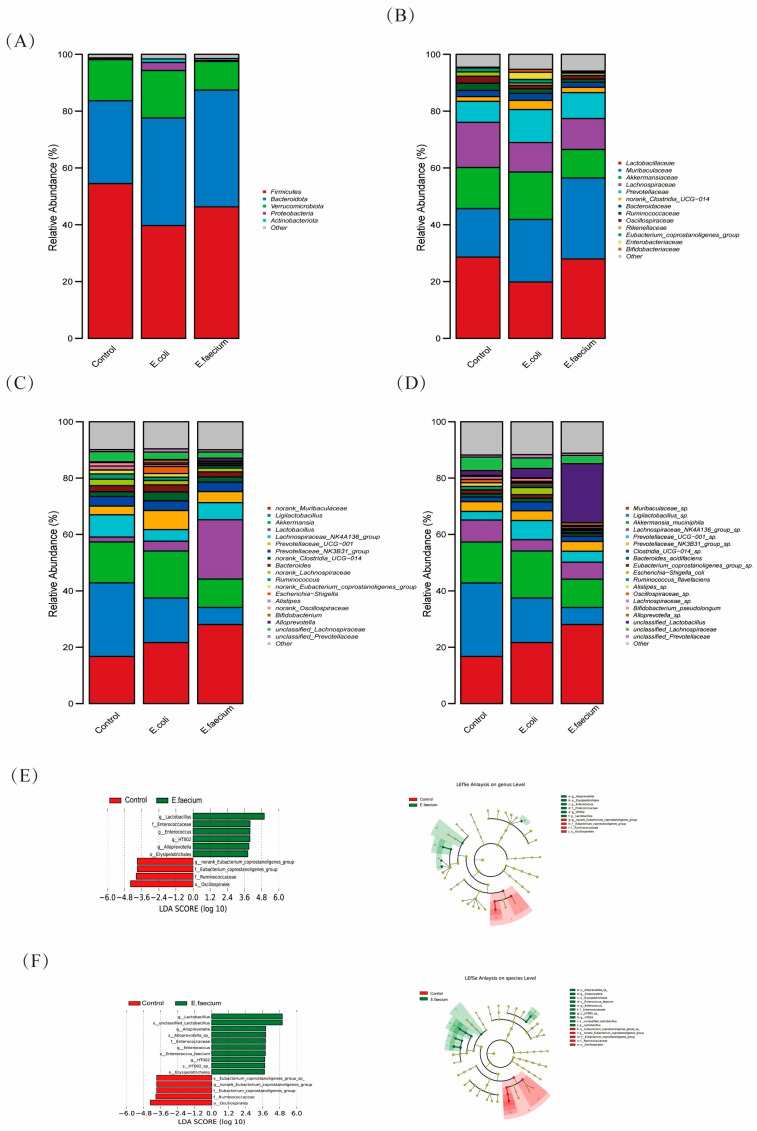
Taxonomic composition of gut microbiota in response to *E. faecium* administration. (**A**) Phylum-level distribution of gut microbiota (**B**), Family-level distribution of gut microbiota (**C**), Genus-level microbial shifts (**D**), Species-level microbial changes (**E**) LEfSe analysis identifying significantly different taxa between groups at the genus and species (**F**) levels, showing taxa with LDA scores > 2.0. Differences in microbial composition reflect the impact of bacterial treatment on gut microbiota structure.

The gut microbiota showed varying relative abundance at different taxonomic levels. At the phylum level, the predominant phyla included Firmicutes, Bacteroidota, *Verrucomicrobiota*, *Proteobacteria*, and *Actinobacteria*. Among these, Firmicutes, Bacteroidota, and *Verrucomicrobiota* were the most abundant. In the Control group, Firmicutes comprised 54.47%, Bacteroidota 29.20%, and Verrucomicrobiota 14.46%. In the *E. coli* group, the relative abundance of Firmicutes was 39.72%, Bacteroidota 37.88%, and Verrucomicrobiota 16.71%. In the E. faecium group, Firmicutes accounted for 46.29%, Bacteroidota 41.13%, and Verrucomicrobiota 10.08%. as shown in Figure 5A. Moreover, family-level analysis results showed that the dominant families included *Lactobacillaceae*, *Muribaculaceae*, *Akkermansiaceae*, *Lachnospiraceae*, *Prevotellaceae*, *norank_Clostridia_UCG-014*, *Bacteroidaceae*, *Ruminococcaceae*, *Oscillospiraceae*, *Rikenellaceae*, *Eubacterium_coprostanoligenes_group*, *Enterobacteriaceae*, and *Bifidobacteriaceae*. *Lactobacillaceae*, *Muribaculaceae*, *Akkermansiaceae*, *Lachnospiraceae*, and *Prevotellaceae* were the most abundant families. In the Control group, *Lactobacillaceae* represented 28.63%, *Muribaculaceae* 17.08%, *Akkermansiaceae* 14.46%, *Lachnospiraceae* 15.89%, and *Prevotellaceae* 7.40%. In the *E. coli* group, *Lactobacillaceae* made up 19.84%, *Muribaculaceae* 22.02%, *Akkermansiaceae* 16.71%, *Lachnospiraceae* 10.36%, and *Prevotellaceae* 11.65%. In the *E. faecium* group, *Lactobacillaceae* accounted for 27.93%, *Muribaculaceae* 28.52%, *Akkermansiaceae* 10.08%, *Lachnospiraceae* 10.87%, and *Prevotellaceae* 9.13% as depicted in Figure 5B.

At the genus level, the predominant genera included *Muribaculaceae*, *Ligilactobacillus*, *Akkermansia*, *Lactobacillus*, *Lachnospiraceae_NK4A136_group*, *Prevotellaceae_UCG-001*, *Prevotellaceae_NK3B31_group*, *Bacteroides*, *Ruminococcus*, and *Escherichia-Shigella*. Notable shifts in the microbiota were observed: *Muribaculaceae*, *Lactobacillus*, and *Alloprevotella* increased in relative abundance, while *Ligilactobacillus*, *Akkermansia*, *Lachnospiraceae*, *Ruminococcus*, and *Eubacterium* groups decreased. The *E. faecium* group showed a marked increase in *Muribaculaceae* and *Lactobacillus*. In contrast, the *E. coli* group showed a moderate shift in the composition without drastic changes in the abundance of these genera (Figure 5C).

At the species level analysis as illustrated in Figure 5D, the predominant species included *Muribaculaceae_*sp., *Ligilactobillustratedacillus_*sp., *Akkermansia_muciniphila*, *Lachnospiraceae_NK4A136_group_*sp., *Prevotellaceae_UCG-001_*sp., *Bacteroides_acidifaciens*, *Escherichia-Shigella_coli*, *Ruminococcus_flavefaciens*, *Alistipes_*sp., and *Oscillospiraceae_*sp. Upon administration of *E. faecium*, the relative abundance of *Muribaculaceae_*sp., *Alloprevotella_*sp., and *unclassified_Lactobacillus* increased significantly, while species such as *Ligilactobacillus_*sp., *Akkermansia_muciniphila*, *Eubacterium_coprostanoligenes_group_*sp., *Alistipes_*sp., and *Oscillospiraceae_*sp. were reduced. The *E. faecium* group showed a notable increase in *Muribaculaceae_*sp. (28.09%) and *unclassified_Lactobacillus* (20.92%), with a concurrent decrease in *Ligilactobacillus_*sp. and other key species. LEfSe analysis (Figure 5E,F) revealed distinct bacterial enrichment among the groups. At the genus level, *E. faecium* increased beneficial taxa such as Lactobacillus and Limosilactobacillus, while *E. coli* elevated Escherichia–Shigella. At the species level, *E. faecium* promoted *L. reuteri* and *L. murinus*, indicating its positive modulation of gut microbiota composition.

### 3.6. Histological Examination

**Figure 6:**
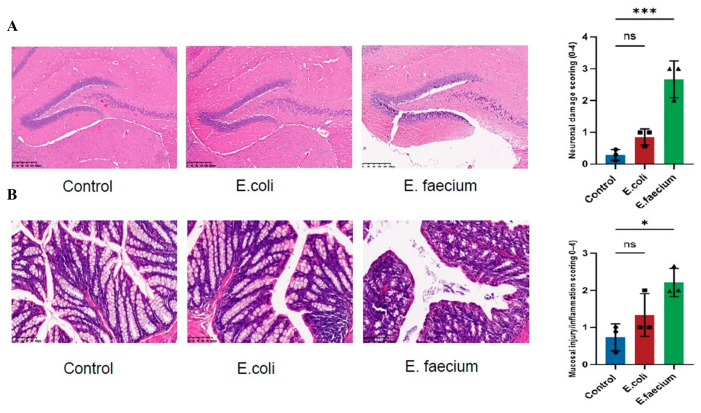
Histological analysis of brain and colon tissues. (**A**) Representative H&E-stained section of the hippocampal dentate gyrus (DG) region showing histopathological alterations in the *E. faecium*-treated group. (**B**) Representative H&E-stained colon tissue showing epithelial damage and inflammatory cell infiltration in the *E. faecium*-treated group. Images captured at 20× magnification; scale bar = 100 µm. Quantitative histological scoring of hippocampal (**A**) and colonic (**B**) tissues. Scoring was performed by two blinded observers using standard damage criteria (brain: 0–3; colon: 0–4). Data represent mean ± SEM (n = 3). Statistical analysis was performed using one-way ANOVA. * *p* < 0.05, *** *p* < 0.001 vs. control; ns, not significant.

To further evaluate the role of *E. faecium* derived from ASD individuals’ a histological examination was conducted using Hematoxylin and Eosin (HE) staining to assess the neuronal morphology. In the control group. The hippocampal dentate gyrus (DG) region exhibited normal neuronal morphology with well-organized cell arrangement, distinct nucleoli, and no evidence of cell shrinkage or necrosis. On the other hand, the *E. coli* group showed no significant changes in the morphology of the hippocampal DG region. However, in the *E. faecium* group, substantial neuronal damage was observed, including nuclear shrinkage, cell body shrinkage and deformation, and extensive necrosis, indicating significant neuronal injury as shown in Figure 6A.

Furthermore, histological analysis of colon tissue revealed differences across groups. In the normal control group, the colon tissue had intact mucosal epithelium and a well-organized glandular structure, with no signs of inflammatory cell infiltration or cell death. The *E. coli* group exhibited no major differences from the control group, although some mild inflammatory cell infiltration was observed in certain areas. In contrast, the *E. faecium* group showed a notable histological damage, inducing disordered glandular arrangement, glandular atrophy, extensive inflammatory cell infiltration, and visible cell death, indicating substantial tissue damage (Figure 6B).

### 3.7. Immunohistochemistry Results

**Figure 7:**
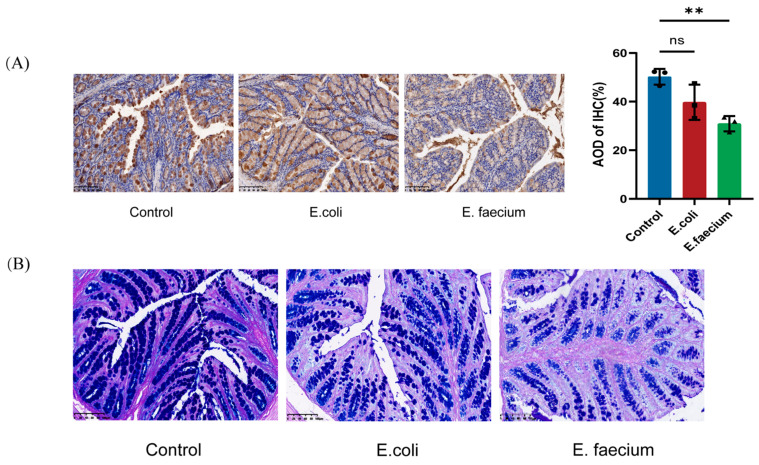
Effect of Enterococcus faecium on intestinal barrier integrity. (**A**) MUC2 expression in the colon shows reduced staining intensity in the *E. faecium* group. (**B**) AB–PAS staining of goblet cells reveals atrophied and decreased goblet cell numbers in the *E. faecium* group. Images captured at 20× magnification; scale bar = 100 µm. ** *p* < 0.01, ns, not significant.

Immunohistochemistry was performed to evaluate the potential impact of *Enterococcus faecium* on the intestinal mucus barrier. Compared to the Control group, the expression of MUC2 in the colon of the *E. coli* group showed a slight, but statistically insignificant, decrease. However, in the *E. faecium* group, there was a significant reduction in both the area and intensity of brown staining in the colon, indicating substantial damage to the intestinal mucus barrier, as illustrated in Figure 7A. AB-PAS staining was employed to examine goblet cell secretion, which reflects the intestinal chemical barrier. The results demonstrate that, in the Control group, goblet cells in the colon were regularly distributed on both sides of the crypt, with well-preserved morphology, deep staining, and abundant mucus. In the *E. coli* group, goblet cells showed similar morphology to the Control group, with no significant changes. In contrast, the *E. faecium* group exhibited atrophied goblet cells that were reduced in number, uneven in size, and displayed lighter staining, suggesting impaired goblet cell function and secretion (Figure 7B).

### 3.8. Western Blotting for Tight Junction Proteins

**Figure 8:**
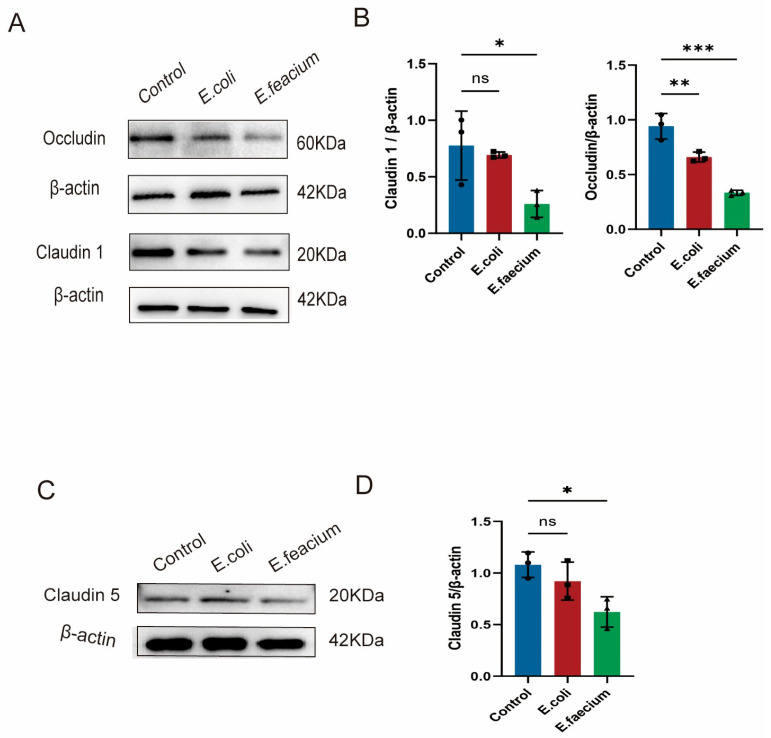
Effect of *E. faecium* on tight junction protein expression in the colon of mice. (**A**) Representative Western blots showing the expression levels of Occludin and Claudin-1 in colonic tissues from control, *E. coli*-treated, and *E. faecium*-treated mice, with β-actin as a loading control. (**B**,**C**) Quantitative analysis of Claudin-1 and Occludin, and (**D**) Claudin-5 normalized to β-actin. Data are presented as mean ± SD (n = 3 per group). Statistical significance was determined by one-way ANOVA followed by Tukey’s post hoc test. * *p* < 0.05, ** *p* < 0.01, *** *p* < 0.001; ns, not significant.

To further investigate the changes in the intestinal mechanical barrier, the expression levels of tight junction proteins Claudin1 and Occludin in colon tissue were measured. Western blotting was employed to quantify the expression of Claudin1 and Occludin in colon tissue, providing an accurate assessment of mechanical barrier integrity. Results found that in the *E. faecium* group, the expression level of Claudin and Occludin was decreased. On the other hand, the *E. coli* group showed a decrease in the expression levels of Occludin but no significant change in the expression of claudin1, as depicted in Figure 8A. Furthermore, the expression level of Claudin5 in cortical tissue was also analyzed using Western blot analysis. Results demonstrated a significant reduction in the expression of Claudin 5 for the *E. faecium* group, whereas no notable changes were observed in the *E. coli* group, as shown in Figure 8B.

### 3.9. Expression of Blood–Brain Barrier and Astrocyte Markers in Brain Tissue

**Figure 9:**
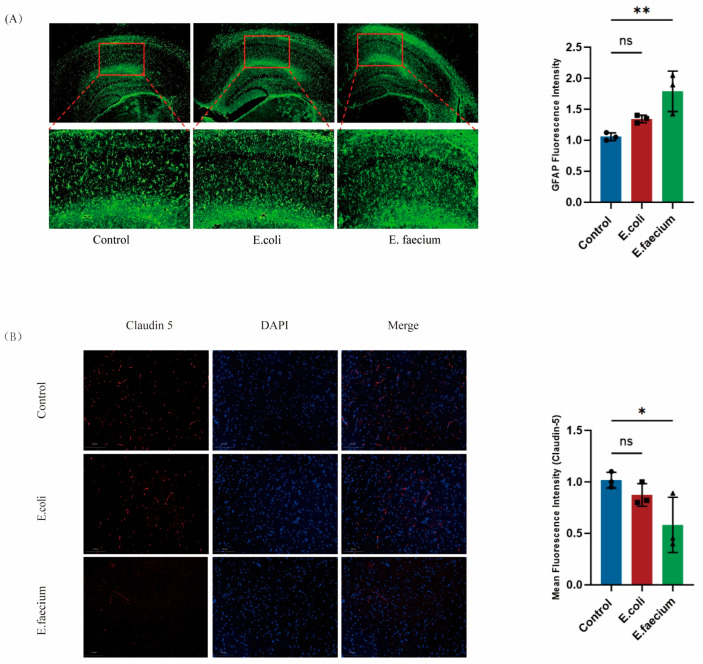
Effect of *Enterococcus faecium* on astrocyte activation and blood–brain barrier integrity. (**A**) Representative GFAP immunofluorescence images in the hippocampus (20×; scale bar = 100 μm) showing marked astrocyte activation in the *E. faecium* group. (**B**) Claudin-5 immunofluorescence staining in cortical tissue (20×; scale bar = 100 μm) demonstrating reduced expression in the *E. faecium* group, indicative of BBB disruption. Quantification was performed from three independent images per sample by two blinded observers. Data are presented as mean ± SEM (n = 3). * *p* < 0.05, ** *p* < 0.01 vs. control; ns, not significant.

To further elucidate the pathogenesis and mechanism of the *E. faecium* derived from ASD patients, the expression level of GFAP, a marker of astrocytes, was determined to determine changes in astrocyte activation and abundance using Immunofluorescence. Results showed a significant increase in the expression of GFAP-positive cells in the hippocampal tissue of the *E. faecium*-treated group compared to the Control group. Additionally, the morphology of the GFAP-positive cells in the *E. faecium*-treated group showed a distinct alteration, including enlargement of the cells’ bodies, a reduction in the length of the processes, and an increase in the number of processes, all indicative of substantial astrocyte activation. In contrast, the *E. coli* group showed no significant differences in GFAP expression or activation compared to the Control group (Figure 9A). furthermore, Claudin-5, a key tight junction protein in the blood–brain barrier integrity, was assessed using immunofluorescence analysis for cortical tissue. Results revealed a marked decrease in the expression of Claudin-5 in the *E. faecium* group, suggesting a disruption in blood–brain barrier function. On the other hand, the *E. coli* group showed similar claudin-5 expression, indicating no significant effect on the integrity of the blood–brain barrier as reflected in Figure 9B.

### 3.10. ELISA Evaluation of Cytokine Levels in Hippocampus and Serum

**Figure 10:**
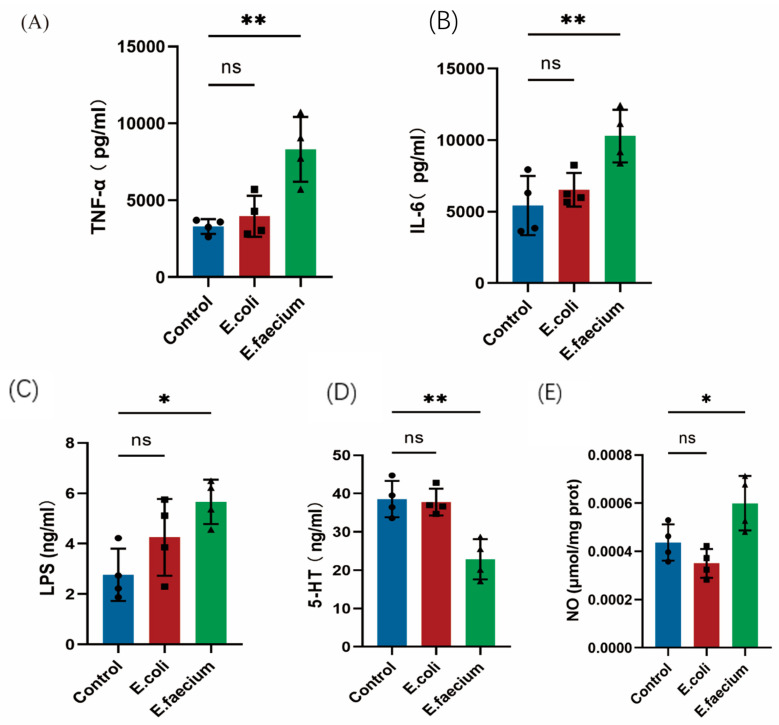
Inflammatory markers and neurotransmitters in response to *E. faecium* treatment. (**A**) TNF-α in the hippocampus (**B**), IL-6 in the hippocampus (**C**), LPS in serum (**D**), 5-HT in serum (**E**) NO in the cortex. * *p* < 0.05, ** *p* < 0.01, ns, not significant.

The expression levels of pro-inflammatory cytokines, including TNF-α and IL-6, in the hippocampus were analyzed using ELISA. The results showed that, compared to the control group, E. faecium demonstrated a significantly higher expression level of TNF-α and IL-6 in the hippocampus, while no significant change was observed in the *E. coli*-treated group (Figure 10A,B). The analysis was further expanded to serum analysis for LPS and 5-HT expression. The results revealed that LPS levels were significantly elevated in the *E. faecium*-treated group in comparison to the normal control group. In contrast, the E. coli group showed a slight, though not significant, increase (Figure 10C). It is important to note that *E. faecium* is a Gram-positive bacterium. It therefore does not produce lipopolysaccharide (LPS). The observed elevation in serum LPS levels in *E. faecium*-treated mice is unlikely to originate directly from this bacterium. Instead, it may reflect secondary effects of gut microbiota dysbiosis, where *E. faecium* colonization disrupts microbial balance, promoting the translocation or overgrowth of Gram-negative bacteria that release LPS into systemic circulation. In addition, 5-HT, an important neurotransmitter that regulates mood, sleep, and appetite, was found significantly elevated in the *E. faecium* group compared to the Control group, with no significant differences in the *E. coli* group, as shown in Figure 10D. Furthermore, the content of NO in the cortex was measured using a micro assay method. The results shown in (Figure 10E) indicated a significant increase in NO levels in the cortex of the *E. faecium* group compared to the Control group, while no significant changes were observed in the *E. coli* group.

## 4. Discussion

Autism Spectrum Disorder (ASD) is a group of neurodevelopmental disorders commonly observed in early childhood. Over the past two decades, the prevalence of ASD has steadily increased, with an estimated incidence of 1 in 36 children [21] and a higher prevalence in males compared to females [22]. Nearly three-quarters of children with ASD also suffer from other medical, psychiatric, or neurological disorders, creating a continuous burden for individuals with autism, their families, and society as a whole [23]. The pathogenesis of ASD remains unclear, and identifying its causative factors and mechanisms is crucial for early intervention and the discovery of selective biomarkers for ASD.

*Enterococcus faecium* is known to acquire various virulence factors and develop resistance to different classes of antibiotics, classifying it as an opportunistic pathogen. As an invasive bacterium, *E. faecium* can penetrate the mucosal epithelium of the gastrointestinal tract and invade host tissues, leading to ectopic colonization in various organs [24]. Recent mechanistic work supports the capacity of *Enterococcus* species to modulate neuroinflammation via the gut–brain axis. For example, *E. faecalis* treatment in an MPTP mouse model of Parkinson’s disease attenuated neuroinflammation, dopaminergic neuronal loss, and blood–brain barrier disruption, with effects dependent on an intact vagus nerve [25,26]. Additionally, gut-derived *Enterococcus* may produce histamine, a neurotransmitter involved in TNF-α-driven neuroinflammatory signaling [27]. Although direct mechanistic studies of *E. faecium* in neuroinflammation are currently limited, these findings highlight the plausibility of our approach and underscore the need for further investigation.

Behavioral experiments are essential in studying the pathogenesis of human diseases. Mouse disease models can simulate human symptoms and explore the underlying pathological mechanisms. The open field test, based on rodents’ natural fear of open spaces and their instinctive preference for sweet food, uses parameters such as total distance traveled, central zone time, and sugar water preference index to assess depression and anxiety in animals [28,29]. Compared to the control group, mice in the *E. faecium* group showed a stronger tendency to remain in the peripheral areas of the open field, with reduced total distance traveled and lower sugar water intake, indicating anxiety and depressive-like behavior. In the Y-maze test, the *E. faecium* group mice showed significantly lower accuracy in exploring different arms, suggesting impaired spatial working memory. The three-chamber social test, a widely used method for evaluating social behavior in rodents [30,31,32], revealed that the *E. faecium* group mice showed a preference for exploring the empty cage, indicating a defect in social interaction.

The gut and its microbiota are implicated in various diseases, and dysbiosis may increase the risk of leaky gut [33,34]. The abundance of *Enterococcus* in *E. faecium* significantly increased, confirming successful colonization in the mouse gut. *Ligilactobacillus*, a beneficial probiotic, plays a key role in antimicrobial activity, immune modulation, and gut microbiota regulation [35,36,37]. *Akkermansiaceae*, a promising next-generation probiotic, offers numerous health benefits, including enhancing intestinal mucosal barrier integrity, controlling body weight, delaying aging, anti-cancer and anti-inflammatory properties, and improving metabolic health [38,39]. *Ruminococcaceae*, an important butyrate-producing bacterium, also has beneficial effects [40,41]. Successful colonization of *E. faecium* can disrupt gut microbial homeostasis, decreasing the abundance of beneficial bacteria like *Ligilactobacillus*, *Akkermansiaceae*, and *Ruminococcaceae*, while increasing harmful bacteria. Known to elevate lactic acid and gastrointestinal symptoms, children with ASD exhibit higher aerobic glycolysis, leading to increased glycolysis, which may be a trigger for ASD [42,43]. The impaired symbiosis between the gut microbiota and gastrointestinal tract may result in bacterial translocation, leading to the transfer of antigens, toxins, or other microbial products from the gut lumen into the bloodstream, triggering intestinal inflammation [44,45].

Histological examination of colon tissues in the Control group revealed a normal structure, characterized by intact mucosal epithelium, orderly gland arrangement, and the absence of inflammatory cell infiltration or cell death. In the *E. coli* group, no significant differences were observed compared to the Control group, but mild inflammatory cell infiltration was noted. The *E. faecium* group showed disorganized gland structure, crypt atrophy, and pronounced inflammatory cell infiltration with cell death, indicating pathological damage. Occludin and Claudin-1, key proteins in the intestinal mechanical barrier, play crucial roles in maintaining intestinal homeostasis [46,47]. Occludin and Claudin-5 are crucial components of tight junction complexes that maintain the integrity of both the intestinal and blood–brain barriers. Reduced expression of these proteins increases permeability and promotes inflammatory infiltration [47]. Immunohistochemistry and Western Blot results showed a significant reduction in the relative expression of both tight junction proteins following *E. faecium* colonization. *E. coli* also significantly decreased Occludin expression, though the decrease in Claudin-1 expression was not significant, suggesting that both bacteria disrupt the intestinal mechanical barrier.

MUC2, the main component of the intestinal mucus layer secreted by goblet cells, covers the apical surface of intestinal epithelial cells, providing structural stability and resistance to the mucus barrier [48,49]. MUC2 expression and goblet cell secretion can reflect the chemical barrier status. Compared to the Control group, goblet cell morphology in the *E. coli* group was unaltered, with slight, but not significant, reductions in MUC2 expression. However, the *E. faecium* group showed atrophy of goblet cells and a significant reduction in both MUC2-positive regions and intensity, indicating damage to the intestinal chemical barrier. Disruption of the intestinal barrier increases gut permeability [50,51,52], allowing bacteria and metabolites to enter the bloodstream. This facilitates antigen exposure to immune cells, breaking immune tolerance and triggering inflammatory responses, which can release pro-inflammatory cytokines [53,54]. Compared to the Control group, mice in the *E. faecium* group showed significantly increased levels of pro-inflammatory cytokines TNF-α, IL-6, and serum LPS, while no significant changes were observed in the *E. coli* group. This suggests that *E. faecium* can increase intestinal permeability, leading to the spread of pro-inflammatory endotoxins like LPS into the bloodstream. Elevated LPS levels can induce inflammation and immune responses, potentially affecting brain function and contributing to psychiatric disorders [55,56].

Astrocytes, diverse glial cells in the central nervous system, provide structural support for neurons, maintain ionic balance, and participate in synaptic function and blood–brain barrier formation [57], playing a key role in CNS diseases [58]. GWAS studies have identified that 65% of autism-associated genes are expressed in astrocytes [59]. The hippocampus is a critical brain structure involved in memory formation, emotional regulation, and other cognitive functions [60]. Moreover, astrocyte activation is a hallmark of neuroinflammatory responses and can exhibit distinct phenotypes: the A1 phenotype, characterized by up-regulation of complement cascade genes and neurotoxic properties, and the A2 phenotype, associated with neurotrophic and repair functions [61]. Compared to the Control group, mice given live *E. faecium* exhibited significantly elevated levels of pro-inflammatory cytokines TNF-α and IL-6 in the hippocampus, while the *E. coli* group showed no significant changes in inflammatory marker expression. These results suggest that *E. faecium* can significantly increase pro-inflammatory cytokine expression in the hippocampus. TNF-α and IL-6 are key mediators of CNS inflammation [62,63]. High levels of LPS and pro-inflammatory cytokines can activate astrocytes, transforming them into a neurotoxic state that promotes neuronal death [64,65]. Astrocytes are crucial for the formation and maintenance of the blood–brain barrier. Under neuroinflammatory conditions, astrocytes can produce nitric oxide (NO) through NF-κB signaling [66,67], Excessive NO production can damage the blood–brain barrier, further exacerbating inflammation [68,69]. Subsequent analysis of astrocytes and the blood–brain barrier in mouse brain tissue revealed that in the presence of high cytokine levels, the *E. faecium* group exhibited a significant increase in the number and activation of astrocytes, elevated NO levels, and a substantial reduction in the relative expression of Claudin-5, a blood–brain barrier-related protein. The *E. coli* group showed no significant differences in these parameters. These findings suggest that *E. faecium* induces brain inflammation, and astrocytes, under the mediation of inflammatory factors, may increase blood–brain barrier permeability through excessive NO production via NF-κB signaling. While the present findings reveal strong associations between *Enterococcus faecium* exposure and behavioral, intestinal, and neuroinflammatory alterations, direct mechanistic causality has not yet been established. Future investigations will employ pharmacological inhibition and microbial rescue experiments to confirm the involvement of the TLR4/MyD88–iNOS signaling cascade. Although increasing evidence suggests that *Enterococcus* species can modulate neuroinflammatory responses, mechanistic validation specific to *E. faecium* remains limited and warrants further study. Moreover, this work utilized a single *E. faecium* isolate; subsequent studies will include multiple ASD-derived strains, heat-killed and metabolite controls, and complementary genetic or pharmacological models to dissect strain-specific and viability-dependent effects. Integration of metabolomic and transcriptomic analyses is also planned to elucidate the molecular interactions linking microbial activity, barrier dysfunction, and neuroinflammation within the gut–brain axis.

## 5. Conclusions

In conclusion, by isolating and culturing gut microbiota from fecal samples of children with ASD, we identified *Enterococcus faecium* as a strain that may influence behavioral outcomes and gut–brain axis function in mice. Colonization with this bacterium was associated with gut microbiota dysbiosis and reduced intestinal barrier integrity. Moreover, *E. faecium* appeared to modulate neuroinflammatory responses and blood–brain barrier permeability, suggesting a potential link between this species and gut–brain axis disruption. While these findings provide valuable exploratory insight into the microbial contribution to ASD-related pathophysiology, further mechanistic and clinical studies are needed to establish causality and generalize these observations to humans.

## Data Availability

The data supporting the findings of this study are available from the corresponding author upon reasonable request.

## References

[1] Loomes R., Hull L., Mandy W.P.L. (2017). What is the male-to-female ratio in autism spectrum disorder? A systematic review and meta-analysis. J. Am. Acad. Child Adolesc. Psychiatry.

[2] Wong N.M., Dipasquale O., Turkheimer F., Findon J.L., Wichers R.H., Dimitrov M., Murphy C.M., Stoencheva V., Robertson D.M., Murphy D.G. (2022). Differences in social brain function in autism spectrum disorder are linked to the serotonin transporter: A randomised placebo-controlled single-dose crossover trial. J. Psychopharmacol..

[3] Chaidez V., Hansen R.L., Hertz-Picciotto I. (2014). Gastrointestinal problems in children with autism, developmental delays or typical development. J. Autism Dev. Disord..

[4] Ding X., Xu Y., Zhang X., Zhang L., Duan G., Song C., Li Z., Yang Y., Wang Y., Wang X. (2020). Gut microbiota changes in patients with autism spectrum disorders. J. Psychiatr. Res..

[5] Oh D., Cheon K.-A. (2020). Alteration of gut microbiota in autism spectrum disorder: An overview. J. Korean Acad. Child Adolesc. Psychiatry.

[6] Sharon G., Cruz N.J., Kang D.-W., Gandal M.J., Wang B., Kim Y.-M., Zink E.M., Casey C.P., Taylor B.C., Lane C.J. (2019). Human gut microbiota from autism spectrum disorder promote behavioral symptoms in mice. Cell.

[7] Fattorusso A., Di Genova L., Dell’Isola G.B., Mencaroni E., Esposito S. (2019). Autism spectrum disorders and the gut microbiota. Nutrients.

[8] Tomova A., Husarova V., Lakatosova S., Bakos J., Vlkova B., Babinska K., Ostatnikova D. (2015). Gastrointestinal microbiota in children with autism in Slovakia. Physiol. Behav..

[9] Wan Y., Zuo T., Xu Z., Zhang F., Zhan H., Dorothy C., Leung T.-F., Yeoh Y.K., Chan F.K., Chan R. (2022). Underdevelopment of the gut microbiota and bacteria species as non-invasive markers of prediction in children with autism spectrum disorder. Gut.

[10] Zou R., Xu F., Wang Y., Duan M., Guo M., Zhang Q., Zhao H., Zheng H. (2020). Changes in the gut microbiota of children with autism spectrum disorder. Autism Res..

[11] Finegold S.M., Dowd S.E., Gontcharova V., Liu C., Henley K.E., Wolcott R.D., Youn E., Summanen P.H., Granpeesheh D., Dixon D. (2010). Pyrosequencing study of fecal microflora of autistic and control children. Anaerobe.

[12] Braniste V., Al-Asmakh M., Kowal C., Anuar F., Abbaspour A., Tóth M., Korecka A., Bakocevic N., Ng L.G., Kundu P. (2014). The gut microbiota influences blood-brain barrier permeability in mice. Sci. Transl. Med..

[13] Zhang H., He K., Zhao Y., Peng Y., Feng D., Wang J., Gao Q. (2025). fNIRS Biomarkers for Stratifying Poststroke Cognitive Impairment: Evidence From Frontal and Temporal Cortex Activation. Stroke.

[14] Fiorentino M., Sapone A., Senger S., Camhi S.S., Kadzielski S.M., Buie T.M., Kelly D.L., Cascella N., Fasano A. (2016). Blood–brain barrier and intestinal epithelial barrier alterations in autism spectrum disorders. Mol. Autism.

[15] Zhuang C., Chen Q., Dou X., Zhang Y., Jin W., Lu X., Wan H., Yu L. (2025). Pathogenic Mechanisms of Cerebral Ischemia and Potential Gut-Brain Axis-Oriented Therapeutic Strategies. Phytomedicine.

[16] Hornuss D., Göpel S., Walker S.V., Tobys D., Häcker G., Seifert H., Higgins P.G., Xanthopoulou K., Gladstone B.P., Cattaneo C. (2024). Epidemiological trends and susceptibility patterns of bloodstream infections caused by *Enterococcus* spp. in six German university hospitals: A prospectively evaluated multicentre cohort study from 2016 to 2020 of the R-Net study group. Infection.

[17] Alatorre-Fernández P., Mayoral-Terán C., Velázquez-Acosta C., Franco-Rodríguez C., Flores-Moreno K., Cevallos M.Á., López-Vidal Y., Volkow-Fernández P. (2017). A polyclonal outbreak of bloodstream infections by *Enterococcus faecium* in patients with hematologic malignancies. Am. J. Infect. Control.

[18] Wang Z., Iida N., Seishima J., Okafuji H., Yutani M., Fujinaga Y., Hashimoto Y., Tomita H., Mizukoshi E., Kaneko S. (2022). Patient-derived *Enterococcus faecium* with inflammatory genotypes promote colitis. J. Gastroenterol..

[19] Woods A., Stirling J., Suvarna S. (2019). Bancroft’s theory and practice of histological techniques. Transm. Electron Microsc..

[20] Kiernan J. (2015). Histological and Histochemical Methods.

[21] Maenner M.J. (2021). Prevalence and characteristics of autism spectrum disorder among children aged 8 years—Autism and developmental disabilities monitoring network, 11 sites, United States, 2018. MMWR Surveill. Summ..

[22] Napolitano A., Schiavi S., La Rosa P., Rossi-Espagnet M.C., Petrillo S., Bottino F., Tagliente E., Longo D., Lupi E., Casula L. (2022). Sex differences in autism spectrum disorder: Diagnostic, neurobiological, and behavioral features. Front. Psychiatry.

[23] Lord C., Bishop S.L. (2010). Autism Spectrum Disorders: Diagnosis, Prevalence, and Services for Children and Families. Social Policy Report. Soc. Res. Child Dev..

[24] Krawczyk B., Wityk P., Gałęcka M., Michalik M. (2021). The many faces of *Enterococcus* spp.—Commensal, probiotic and opportunistic pathogen. Microorganisms.

[25] Shao X., Wu T., Li M., Zheng M., Lin H., Qi X. (2025). *Enterococcus faecalis* Exerts Neuroprotective Effects via the Vagus Nerve in a Mouse Model of Parkinson’s Disease. Mol. Neurobiol..

[26] Luo H., Gu X., Tong G., Han L. (2022). Research progress of apelin in acute ischemic brain injury. Am. J. Transl. Res..

[27] Mejía-Granados D.M., Villasana-Salazar B., Coan A.C., Rizzi L., Balthazar M.L.F., de Godoi A.B., do Canto A.M., Silva L.S., do Rosario Tacla R., Damasceno A. (2022). Gut microbiome in neuropsychiatric disorders. Arq. Neuro-Psiquiatr..

[28] Neelotpol S., Rezwan R., Singh T., Mayesha I.I., Saba S., Jamiruddin M.R. (2024). Pharmacological intervention of behavioural traits and brain histopathology of prenatal valproic acid-induced mouse model of autism. PLoS ONE.

[29] Du Y., Chen L., Yan M.-C., Wang Y.-L., Zhong X.-L., Xv C.-X., Li Y.-B., Cheng Y. (2023). Neurometabolite levels in the brains of patients with autism spectrum disorders: A meta-analysis of proton magnetic resonance spectroscopy studies (N = 1501). Mol. Psychiatry.

[30] Szabó J., Renczés E., Borbélyová V., Ostatníková D., Celec P. (2024). Assessing sociability using the Three-Chamber Social Interaction Test and the Reciprocal Interaction Test in a genetic mouse model of ASD. Behav. Brain Funct..

[31] Guo P., Zeng M., Liu M., Zhang Y., Jia J., Zhang Z., Liang S., Zheng X., Feng W. (2025). Zingibroside R1 Isolated From Achyranthes bidentata Blume Ameliorates LPS/D-GalN-Induced Liver Injury by Regulating Succinic Acid Metabolism via the Gut Microbiota. Phytother. Res..

[32] Liang J., Chen L., Li Y., Chen Y., Yuan L., Qiu Y., Ma S., Fan F., Cheng Y. (2024). Unraveling the prefrontal cortex-basolateral amygdala pathway’s role on schizophrenia’s cognitive impairments: A multimodal study in patients and mouse models. Schizophr. Bull..

[33] Qiu P., Ishimoto T., Fu L., Zhang J., Zhang Z., Liu Y. (2022). The gut microbiota in inflammatory bowel disease. Front. Cell. Infect. Microbiol..

[34] Ristori M.V., Quagliariello A., Reddel S., Ianiro G., Vicari S., Gasbarrini A., Putignani L. (2019). Autism, gastrointestinal symptoms and modulation of gut microbiota by nutritional interventions. Nutrients.

[35] Guerrero Sanchez M., Passot S., Campoy S., Olivares M., Fonseca F. (2022). Ligilactobacillus salivarius functionalities, applications, and manufacturing challenges. Appl. Microbiol. Biotechnol..

[36] Mukohda M., Yano T., Matsui T., Nakamura S., Miyamae J., Toyama K., Mitsui R., Mizuno R., Ozaki H. (2023). Treatment with Ligilactobacillus murinus lowers blood pressure and intestinal permeability in spontaneously hypertensive rats. Sci. Rep..

[37] Tang L., Wang Y., Gong X., Xiang J., Zhang Y., Xiang Q., Li J. (2023). Integrated transcriptome and metabolome analysis to investigate the mechanism of intranasal insulin treatment in a rat model of vascular dementia. Front. Pharmacol..

[38] Liu C., Gong J., Zhang Q., Chen G., Yin S., Luo Z., Zeng W., Yu J., Lan P., He Z. (2023). Dietary iron modulates gut microbiota and induces SLPI secretion to promote colorectal tumorigenesis. Gut Microbes.

[39] Xie S., Li J., Lyu F., Xiong Q., Gu P., Chen Y., Chen M., Bao J., Zhang X., Wei R. (2024). Novel tripeptide RKH derived from Akkermansia muciniphila protects against lethal sepsis. Gut.

[40] Wang L., Liao Y., Yang R., Zhu Z., Zhang L., Wu Z., Sun X. (2021). An engineered probiotic secreting Sj16 ameliorates colitis via Ruminococcaceae/butyrate/retinoic acid axis. Bioeng. Transl. Med..

[41] Darnaud M., Dos Santos A., Gonzalez P., Augui S., Lacoste C., Desterke C., De Hertogh G., Valentino E., Braun E., Zheng J. (2018). Enteric delivery of regenerating family member 3 alpha alters the intestinal microbiota and controls inflammation in mice with colitis. Gastroenterology.

[42] Rossignol D., Frye R.E. (2012). Mitochondrial dysfunction in autism spectrum disorders: A systematic review and meta-analysis. Mol. Psychiatry.

[43] Vallée A., Vallée J.-N. (2018). Warburg effect hypothesis in autism Spectrum disorders. Mol. Brain.

[44] Lechuga S., Naydenov N.G., Feygin A., Cruise M., Ervasti J.M., Ivanov A.I. (2020). Loss of β-cytoplasmic actin in the intestinal epithelium increases gut barrier permeability in vivo and exaggerates the severity of experimental colitis. Front. Cell Dev. Biol..

[45] Agus A., Planchais J., Sokol H. (2018). Gut microbiota regulation of tryptophan metabolism in health and disease. Cell Host Microbe.

[46] Pan Y., Ning Y., Hu J., Wang Z., Chen X., Zhao X. (2021). The preventive effect of lactobacillus plantarum ZS62 on DSS-Induced IBD by regulating oxidative stress and the immune response. Oxidative Med. Cell. Longev..

[47] Bhat A.A., Uppada S., Achkar I.W., Hashem S., Yadav S.K., Shanmugakonar M., Al-Naemi H.A., Haris M., Uddin S. (2019). Tight junction proteins and signaling pathways in cancer and inflammation: A functional crosstalk. Front. Physiol..

[48] Yao D., Dai W., Dong M., Dai C., Wu S. (2021). MUC2 and related bacterial factors: Therapeutic targets for ulcerative colitis. EBioMedicine.

[49] Paone P., Cani P.D. (2020). Mucus barrier, mucins and gut microbiota: The expected slimy partners?. Gut.

[50] Wang J., He M., Yang M., Ai X. (2024). Gut microbiota as a key regulator of intestinal mucosal immunity. Life Sci..

[51] Glymenaki M., Singh G., Brass A., Warhurst G., McBain A.J., Else K.J., Cruickshank S.M. (2017). Compositional changes in the gut mucus microbiota precede the onset of colitis-induced inflammation. Inflamm. Bowel Dis..

[52] Chen X., Shi S., Sun C., Li S. (2024). A study of the relationship between inflammatory immune function and intestinal Flora in adolescent patients with first-episode depression. Actas Españolas Psiquiatr..

[53] Takiishi T., Fenero C.I.M., Câmara N.O.S. (2017). Intestinal barrier and gut microbiota: Shaping our immune responses throughout life. Tissue Barriers.

[54] Wu S., Fang X., Zhao J., Liu G., Liao P. (2025). Nutrient regulation targeting macrophage-controlled intestinal mucosal healing: A promising strategy against intestinal mucositis induced by deoxynivalenol. Toxicon.

[55] Qin L., Wu X., Block M.L., Liu Y., Breese G.R., Hong J.S., Knapp D.J., Crews F.T. (2007). Systemic LPS causes chronic neuroinflammation and progressive neurodegeneration. Glia.

[56] Cheng X., Tan Y., Li H., Huang J., Zhao D., Zhang Z., Yi M., Zhu L., Hui S., Yang J. (2022). Fecal 16S rRNA sequencing and multi-compartment metabolomics revealed gut microbiota and metabolites interactions in APP/PS1 mice. Comput. Biol. Med..

[57] Jiang J., Zhang L., Wu D., Zhao D., Ying S., Ding S. (2025). Lipopolysaccharide induces neuroinflammation in a valproic acid male model of autism. Brain Res. Bull..

[58] Lee H.-G., Wheeler M.A., Quintana F.J. (2022). Function and therapeutic value of astrocytes in neurological diseases. Nat. Rev. Drug Discov..

[59] Timothy W.Y., Chahrour M.H., Coulter M.E., Jiralerspong S., Okamura-Ikeda K., Ataman B., Schmitz-Abe K., Harmin D.A., Adli M., Malik A.N. (2013). Using whole-exome sequencing to identify inherited causes of autism. Neuron.

[60] Kuijer E.J., Steenbergen L. (2023). The microbiota-gut-brain axis in hippocampus-dependent learning and memory: Current state and future challenges. Neurosci. Biobehav. Rev..

[61] Liddelow S.A., Guttenplan K.A., Clarke L.E., Bennett F.C., Bohlen C.J., Schirmer L., Bennett M.L., Münch A.E., Chung W.-S., Peterson T.C. (2017). Neurotoxic reactive astrocytes are induced by activated microglia. Nature.

[62] Linnerbauer M., Wheeler M.A., Quintana F.J. (2020). Astrocyte crosstalk in CNS inflammation. Neuron.

[63] Zhong S., Sun Z., Tian Q., Wen W., Chen F., Huang X., Li Y. (2024). Lactobacillus delbrueckii alleviates lipopolysaccharide-induced muscle inflammation and atrophy in weaned piglets associated with inhibition of endoplasmic reticulum stress and protein degradation. FASEB J..

[64] Lee K.-S., Yoon S.-H., Hwang I., Ma J.-H., Yang E., Kim R.H., Kim E., Yu J.-W. (2024). Hyperglycemia enhances brain susceptibility to lipopolysaccharide-induced neuroinflammation via astrocyte reprogramming. J. Neuroinflammation.

[65] Huang L., Tan J., Lin P., Chen Z., Huang Q., Yao H., Jiang L., Long B., Long Y. (2024). Autoimmune encephalitis followed by hemophagocytic lymph histiocytosis: A case report. Front. Immunol..

[66] Wheeler M.A., Jaronen M., Covacu R., Zandee S.E., Scalisi G., Rothhammer V., Tjon E.C., Chao C.-C., Kenison J.E., Blain M. (2019). Environmental control of astrocyte pathogenic activities in CNS inflammation. Cell.

[67] Zhang Y., Deng J., Chen T., Liu S., Tang Y., Zhao J.R., Guo Z., Zhang W. (2024). Formononetin alleviates no reflow after myocardial ischemia-reperfusion via modulation of gut microbiota to inhibit inflammation. Life Sci..

[68] Calabrese V., Mancuso C., Calvani M., Rizzarelli E., Butterfield D.A., Giuffrida Stella A.M. (2007). Nitric oxide in the central nervous system: Neuroprotection versus neurotoxicity. Nat. Rev. Neurosci..

[69] He Y., Hu H., Liang X., Liang J., Li F., Zhou X. (2025). Gut microbes-muscle axis in muscle function and meat quality. Sci. China Life Sci..

